# A queer feminist posthuman framework for bioethics: on vulnerability, antimicrobial resistance, and justice

**DOI:** 10.1007/s40592-024-00192-4

**Published:** 2024-11-13

**Authors:** Tiia Sudenkaarne

**Affiliations:** Department of Sociology, University of Helsinki, Helsinki, Finland

**Keywords:** Vulnerability, Queer bioethics, Feminist bioethics, Antimicrobial resistance, Posthumanism

## Abstract

In this paper, I discuss the bioethical principle of justice and the bioethical key concept of vulnerability, in a queer feminist posthuman framework. I situate these contemplations, philosophical by nature, in the context of antimicrobial resistance (AMR), one the most vicious moral problems of our time. Further, I discuss how gender and sexual variance, vulnerability and justice manifest in AMR. I conclude by considering my queer feminist posthuman framework for vulnerability and justice in relation to the notion of antibiotic vulnerabilities, suggesting a lacuna for further AMR research.

## Introduction

The concept of vulnerability first emerged into bioethics from research ethics in the 1970s that often labeled certain subpopulations—for example, (cis)women—as vulnerable, without being very resourceful in solving ethical issues stemming from that vulnerability. In recent decades, however, vulnerability has been reconfigured in bioethical theory. The rigid understanding that Florencia to Luna (2009; 2018; Luna and Vanderpoel 2013) calls the subpopulation approach has been challenged with morally more salient approaches, contributing new ideas to debates about the ethical dimensions of medicine and health care (ten Have 2016). Vulnerability cannot be fully understood within the framework of individual autonomy, which can dominate mainstream bioethics; rather, vulnerability is perceived as created through the social and economic conditions of life (ten Have 2016; Rogers et al. 2012; Lange et al. 2013; Macklin 2012). But should that be multispecies life?

To be explained in more detail in another contribution in this special issue, suffice is to say that to recalibrate the posthumanist momentum of interrogating human (see Sudenkaarne and Butcher 2024) exceptionalism (Wienhues 2017; Boetzkes and Robert 2000) – of conversations around life, subjectivity, human nature, responsibility, and interspecies relations that underpin bioethical decision-making (Sands 2022, 1) – I wish to build on the concept of more-than-human (Cañada and Venäläinen 2022; Whatmore 2006). It is undoubtedly still anthropocentric in the sense that it has human as one of its denominators, but by the idea of *more than*, I not only wish to imply moral significance in the ecocentric and biocentric sense but also to include, in a network theory rather than confessional understanding, spectral realms such as ancestors, spirits, ghosts and deities that are crucial actors in several global sense-making systems (de la Cadena 2010). Making further way for the radical reorientation of human exceptionalism, by *more than* I wish to remain open to finding new ways of being with ethics that perhaps could be more attuned to planetary or even cosmological flourishing than current human-oriented approaches. *More-than-human* interrogates anthropocentrism in moral significance and ethical analyses but does not wish to abandon or exceed the human per se in a transhumanist understanding (Whatmore 2006; Braidotti 2021). It is crucial to note than in this framework, justice and vulnerability are neighboring concepts: in analyses of injustice, on many occasion it is precisely the detection and eradication of vulnerabilities that will pave the way to more just worlds. The intersection of vulnerability and justice opens up a possibility to learn about the suffering of others and recalibrate your moral compass; thus, it also has the possibility to deliver *more than* privileged humans currently fathom or care for.

## A layered approach to vulnerability

From the inception of the field, bioethics has set out to protect vulnerable patients and research subjects from harm, and to establish their moral and legal rights (Holmes 1999, 48; Wolf 1996). According to a persistent line of critique, however, gender-related inequalities, cis- and heteronormativity, and biases based on race/ethnicity, class, and ability continue to go unacknowledged (Lindemann 2007; Nelson 2012; Stramondo 2016; Ray 2020). Feminist bioethics, emerging from women’s health movements, feminist movements and feminist academics, is a polyphonic approach that looks at biomedical issues targeting women and female-gendered health, overlapping with feminist critiques of science, medicine, rationality, and agency (Donchin and Purdy 1999; Rogers et al. 2023). Often joining forces but sometimes also in juxtaposition to a feminist bioethical stance, queer and LGBT bioethics refer to biomedical issues raised by gender and sexual variance, for example challenging the definition of homosexuality or transgender as pathologies, directly accreditable to LGBTQI+ activism (Nelson 1998, 2012; Wahlert and Fiester 2012; Drescher 2015). However, feminist or queer bioethics are not distinctive approaches because they focus the viewpoint of women or queer people, but because they offer unique theoretical and methodological contributions. This also grounds the project of queer feminist moral theory, featuring the necessary condition of gender and sexual variance as opposed to cis- and heteronormativity (Sudenkaarne 2020a; 2021). Cis- and heteronormativity refers to the ethos of labelling existence – bodies but also reality per se – into binary, juxtaposing categories that through stereotyping (e.g. gender roles) harshly limit human flourishing. To decolonial feminism philosopher Maria Lugones (2007, 202), cis- and heteronormativity continue to be downplayed despite its “consistently perverse, violent, and demeaning effect, turning people into animals and turning white women into reproducers of the (white) race and the (middle or upper) class” (see also Sudenkaarne and Blell 2022). This suggests a morally salient yet volatile nexus between a multispecies approach, justice orientations, decolonial framework, class politics and a queer feminist posthumanist framework, most of these aspects to be considered elsewhere. What I argue in this paper is that vulnerability and justice are excellent conceptual tools to address phenomena like AMR that include all of these nexae. However, both concepts require new frameworks.

For Becky Holmes (1999, 49–53), the long-standing disempowerment of patients despite bioethics’ long-standing concern about vulnerable populations begs the question of whether bioethics’ alliance with institutionalized power keeps it closed to issues that concern so-called marginalized groups. It can be argued, however, that the vague formulation of such critiques, which target a generalization of “bioethics,” makes it difficult to address and resolve such issues. What I suggest is the formulation of a queer feminist framework for reconfiguring key bioethical concepts. In my analysis, vulnerability has become central (Sudenkaarne 2021), which is what I will focus on next. Moreover, however, a queer feminist framework should guide the reconfiguration of bioethical principles, which I will discuss later.

Although marginalized groups have unique standpoints, those views often get eliminated from conversation as nonuniversal. For some feminist critics, it is the deeply structured embrace of liberal individualism that obscures the importance of groups (Holmes 1999, 53). In this line of thinking, social groups are tools of oppression: one’s identity can make one particularly vulnerable to cultural imperialism (including the stereotyping, erasure, or appropriation of one’s group identity), violence, exploitation, marginalization, or powerlessness. What becomes crucial about the “identity” in identity politics in this context appears to be the experience of the subject; yet that experience is shaped by memberships of social groups (Heyes 2020). These groups are surely important and necessary for self-esteem, physical and emotional survival, and the joys and pleasures of life (Holmes 1999, 58). But when certain groups are marginalized, does every member of that group also get marginalized? For Holmes (ibid.), if bioethics focuses on individual rights, bioethics may exacerbate group marginalization. This is also pivotal to Anne to Donchin and Laura to Purdy (1999, 8–9), who see power and particularity, the very dynamics of grouping, as dominant in feminist critiques: the powers that divide and marginalize nondominant people, and the particularities of personal lives that resist confinement within externally imposed categories.

The notion of marginalized groups and vulnerability ushers what I have called the problems of essentialism, identity, and relativism into bioethics. If for example LGBTQI + people are considered particularly vulnerable, what about intersectional differences in affluence and access to health care? How can one remain a powerful subject and yet be considered vulnerable? How are we to use vulnerability as a tool for ethical analysis? I wish to next discuss how the reconfigured notion of vulnerability is indeed a useful concept for feminist and queer bioethics, as it can offer a salient way to solve the problems of identity, essentialism, and relativism.

An excessively broad use of the concept of vulnerability renders it too nebulous to be meaningful, and has a stereotyping effect. Luna (2018, 1–2) distinguishes between two spheres in vulnerability debates: conceptually, how vulnerability should be understood; and practically, how it should be accurately used. To answer the conceptual question, she (2009) has reconfigured vulnerability for use as a layered concept. Her approach to vulnerability has been notably accepted and applied (e.g. Rogers et al. 2012; Lange et al. 2013; Macklin 2012).

For Luna (2009, 122), the reconfiguration of vulnerability is motivated by the previously mentioned realization that vulnerability has not been favorable to people considered vulnerable. Her reconfigurations stem from feminist analyses and look at vulnerability as a concept of special interest to women. In research ethics, women are sometimes considered a vulnerable group, and at other times are removed from such a group. For Luna, labeling women or any group simply as vulnerable is too simplistic and is a potential source of grave moral harm if we perceive vulnerability in terms of “being vulnerable”—for example, women being essentially vulnerable, rather than being rendered vulnerable in certain conditions with certain resources—so that vulnerability becomes a fixed label attached to certain subpopulations. This includes the assumption that there are necessary and sufficient conditions that populations must fulfill to be considered vulnerable. When vulnerability is used as a fixed label for a particular subpopulation, it suggests a simplistic answer to a complicated problem. To address the subject’s vulnerability, more than one answer may be needed. Different types of vulnerabilities can overlap, and they should all be adequately considered. Finally, yet importantly, labeling fixes content, and labels do not come off easily (ibid., 123–124; cf. ten Have 2016).

For Luna (2009, 128), then, it becomes necessary to provide an analysis of vulnerability that does not render it vacuous, rescues its force, and avoids some of the morally gravest flaws of labeled vulnerability. She suggests an ethically more sustainable, humanely robust, and pragmatically useful concept of vulnerability as layered. The metaphor of a layer gives the idea of relationality, of something that may be multiple and different but also overlapping: some overlapping layers may be related to health, and others to work, yet both are connected. For example, it can be said that being a woman does not per se imply that a person is vulnerable, but in a country that is intolerant of women’s reproductive rights, a woman acquires that layer of vulnerability. If she is poor and illiterate, she has two more layers of vulnerability. Therefore, we should not think that someone is vulnerable, but instead should consider a particular situation that renders someone vulnerable, which does not mean a categorical lack of power (ibid., 129).

Luna’s (ibid., 134) layered understanding of vulnerability challenges idealized views of the neoliberal subject and agency, since the most serious shortcoming of the rigid vulnerability approach is to treat vulnerability as a label affixed to a particular subpopulation. In Luna and Vanderpoel’s (2013, 326) account, to target subpopulations with a labeling strategy is to assume a baseline standard, or a paradigmatic subject: a mature, moderately well-educated, clear-thinking, literate, self-supporting person. Further, the subpopulation approach assumes the possibility of identifying vulnerabilities in subpopulations as variations from the paradigm. A consequence of the categorical model is a simplistic answer to a complicated problem, as a person or group of persons can suffer differ kinds of vulnerabilities. The label approach understands vulnerability as targeting a permanent and categorical condition that will persist throughout the person’s existence. Thus, subpopulation analysis can lead to an overly rigid and fixed perspective. In contrast, if vulnerability is viewed as layered, there is no single feature that in and of itself defines vulnerability, no solid and unique vulnerability that will exhaust a category, and most importantly, no single feature that can suffice to explain it entirely (ibid.).

I suggest that reconfigured vulnerability offers a way to consider the material bearings of race, gender, and sexuality while also encompassing situated, contextual differences in experiences of them. Such a reconfigured analysis aims to detect risks and harms that are not causal but probable in varying degrees. Layered vulnerability offers an escape from the looping problems of identity, essentialism, and relativism. Instead, it creates a bioethical approach that identifies, evaluates, removes, and alleviates layers of vulnerabilities in a particular context. Luna urges us to consider the dispositional structure of layers of vulnerability, and to assess what stimulus conditions can trigger them (their presence and their probability of developing). Stimulus conditions relate the layers and context with actual conditions and possibilities of occurrence. If the stimulus conditions are highly probable, they should take priority. These conditions are those that actualize the layer of vulnerability and will provoke actual harm. Following on from this identification and evaluation is the obligation to not worsen the person’s or group’s vulnerability, to try to eliminate their layers of vulnerability, or at the very least to minimize the layers^1^ (Luna 2018, 7–8). I will next move to discuss justice as a bioethical principle in relation to vulnerability and AMR, further suggesting a queer feminist posthuman framework.

## Queer feminist posthuman framework for principles: justice and AMR

Familiarly to a reader in bioethics, an approach that utilizes abstract principles to identify and reflect on moral problems is often called principlism. Principles can either be derived from moral theory or not; if not, they are often justified by so-called common morality (Ashcroft et al. 2007; Arras 2016). The common-morality approach by Tomas L. Beauchamp and James F. Childress (2013; see also 2019) is influential both in theory and practice. They define the most commonly used four principles of (respect for) autonomy, a norm respecting and supporting autonomous decision-making; nonmaleficence, a norm avoiding the causation of harm; beneficence, a group of norms pertaining to the relief, lessening, or prevention of harm, the provision of benefits, and the balancing of benefits against risks and costs; and justice, a group of norms to fairly distribute benefits, risks, and costs (Beauchamp and Childress 2013, 13). These abstract principles are accompanied by rules or other elements to inform moral contemplation (Beauchamp and Childress 2013; Ashcroft et al. 2007; Arras 2016).

One of the key queer feminist critiques towards principlism is that common morality reproduces moral objectivity as an unsituated rationality (Tong 1996, 69), or Jose to Canada, Butcher and Sariola (2022), problematic universalism. Such objectivity is biased toward the moral realities and views of those with more social power. Further, unsituatedness is a metaphysical and ethical stance leading to atomistic understanding of autonomy, to which a more relational approach has been suggested elsewhere (Sudenkaarne 2021; see also Donchin 2001). Another key problem is the relationship between common morality and moral theory, which also affects the interpretation and contextualization of the principles. As Rosemary to Tong (1996, 69) observes, even though common-morality approaches often reject moral theory, often with the good intention of providing definitions uncomplicated enough for all stakeholders to access swiftly, they often end up referring to it anyway: beneficence is often drawn from Mill, autonomy from Kant and Mill, justice from Rawls, and nonmaleficence from Gert (cf. Clouser and Gert 1990, 223; see also Donchin 2001, 366). Principlist approaches that do derive from moral theory tend to similarly mix and match, to Tong (ibid.) “presenting the resultant blend as an integrated and unified theory whereas nothing could be further from the truth”.

 I concur that, a defining problem with principlism is its confused relation to moral theory. Adding to the confusion, for example Beauchamp and Childress do sometimes refer to their prominent account of ethics as a theory but deny that it is an integrated body of moral norms or a systematic justification of basic moral norms (2019, 351). Yet whether or not a common-morality view can escape the latter can be contested. Indeed, theories evoking common morality can be seen to constitute a category of common-morality theory (cf. Holm 1995; Arras 2016). Somewhat acknowledging this, Beauchamp and Childress (2013, 422–423; see also 2019) refer to the problems of common-morality theory, leaving, however, “its specification, judgment, coherence, and construction” for further research. Even though these issues might be exhausted from several avenues—for example, by borrowing from virtue ethics, as Beauchamp and Childress most eagerly suggest, or from obligations ─ none of the prominent solutions can resolve the fundamentally confused relation to moral theory without admitting that principlism is a moral theory, in which case it becomes internally confused as a moral theory that borrows from various approaches while fully adhering to none. Further, acknowledging the critical work of feminist philosophy regarding how the premises of Western philosophy are built on gendered, racialized, binary, and hierarchical concepts (Lloyd 1984; Code 2007; Lugones 1994; Anzaldúa 1999), I suggest the formulation of a queer feminist framework, to be further developed into a background theory, might offer reliable responses.

But, as Tong (2013, 29) asks, is there any single set of ethical principles or human values that exists independently of the cultural context in which it has been produced? Probably not, she (ibid.) answers, “unless one is committed to the view that ethical principles are generated in some metaphysical realm prior to the beginning of temporality and spatiality”. Alternatively, are there certain guiding human values, such as human flourishing or the meeting of people’s needs, that emerge in any and all sustainable contexts? Again, following Tong (2013), probably there are. But even if such ethical commonalities exist, their existence in no way guarantees their uniform interpretation, let alone their actual instantiation (Tong 2013, 29). Ethical theories must hence include critical lenses clear enough to recognize the unjust power relations that result in wrongful distributions of limited resources, and a motivational force strong enough to prompt people who currently benefit from unjust power relations to renounce them (Tong 2013, 30). For one of such lenses, I offer a more-than-human justice orientation, as part of a wider queer feminist posthumanist framework.

For many critics, the problem with principlism is not the employment of principles as the units of moral analysis, but the abuse of them. Autumn to Fiester (2015, 310) has identified it as severe curtailment of the set of pertinent ethical concepts. Exclusion and marginalization are forms of this abuse, as are all processes that render the socially less powerful in a moral conflict as unintelligible or indeed immoral; for example, clinical ethical conflicts that appear wildly imbalanced morally can result from unrecognized principles, yet principles can also be weaponized against the morally vulnerable (Fiester 2015, 310). Fiester (ibid.) describes how weaponization begins with the false assumption that all of the pertinent principles and legitimate moral considerations have already been articulated and are limited in number. In a resulting principlist paradigm, they operate like a diagnostic checklist that scans for a handful of ethical considerations, normative assessment basing entirely on that reductive set of ethical concerns (ibid.). This is principlism at its worst, and it is in fact not compatible with Beauchamp and Childress’s approach. Still, such poor ethical analysis can inadvertently weaponize the principles it does recognize, to the detriment of causes and claims anchored by the principles it does not recognize (Fiester 2015, 311). To offer more ethical dexterity to a principlist framework, I shall next briefly introduce feminist and queer bioethics in more detail to enrich it, also serving as a framework for a more-than-human justice orientation.

Feminist bioethics emerged during the 1990s as a distinctive academic focus, offering insights into how gender, ethnicity, and power disparities operate,influenced by as mentioned, the late 20th -century women’s health movement and the early years of second-wave feminism (Donchin and Scully 2015). Activist feminists directed attention to areas of health care where women’s interests were the most obvious but the most severely neglected: access to birth control and abortion, pregnancy, and the control of representations of female sexuality. Feminists campaigned on clinical issues with direct relevance to women’s biology, for instance, increased research into breast cancer. These groups and movements struggled to raise public awareness of women’s health issues, influence national health policies, and act as a counterbalance to the priorities of professional medicine and the pharmaceutical industry. (Donchin and Scully 2015; see also Donchin and Purdy 1999; Holmes 1999; Wolf 1996.) On a moral theoretical level, one of the unique contributions of feminism has been to find gender bias in the preoccupations, assumptions, and perspectives of the dominant theories in Anglo-American ethics (Lindemann 2007, 117). Even though to some critics care as a conceptual tool for analysis and theory-building is too central in feminist bioethics – reproducing caring as essential to women and women essentially as carers – (Lindemann 2007, 117; Held 2005; Carse and Nelson 1996; Tong 2013), care and other medical practice should persist a particular concern for feminists because it is one of the hegemonic discourses of our time, commanding enormous amounts of prestige and authority, in its complex and multiple interactions with gender (Lindeman 2009, 122). Further, such moral theoretical concerns should include a position building on non-binary understanding of gender while still being able to compute gendered concerns associated with both female and male fluxes, in an intersectional setting.

What I suggest, then, is a principlist approach within a queer feminist framework. One of the key justifications for such an endeavor is the omnipresent injustice in various discourses around human ways of being, thinking and doing, and the centrality of justice available in a feminist agenda, to be joined forces with queer and posthumanist components. Donchin and Purdy (1999, 2–3) urge the adoption of what they call core feminism – at the core being various justice-seeking orientations building from lived intersections of injustices and oppressions – for practical, political, and philosophical reasons. The pragmatics is this simple message: justice requires the eradication of inequality. This emphasis is an open call to join forces with other justice movements. The politics of core feminism includes staying open to new ideas from lived lives, leaving room to disagree, and being open to difference, both crucial for a more-than-human understanding of ethics to follow. To Donchin and Purdy (1999, 4) staying open safeguards against “false universalization” – unsituated rationality (Tong 1996) or problematic universalism (Cañada, Sariola and Butcher 2022) – which, however, needs to be developed further with a queer bioethical orientation.

To its developers Lance Wahlert and Autumn Fiester (2012, ii; 2014), queer bioethics, on one hand, focuses on LGBTQI+-specific questions, interrogating how and why gender and sexuality are produced and reproduced, and critically deconstructing them with the analytical tools of cis- and heteronormativity. On the other, it also interrogates why and explains how questions of gender and sexuality are questions of humanity per se (Wahlert and Fiester 2012, ii), and life as we (think we) know it. As recent scientific advances have broadened understanding of, for example, the nonbinary number of chromosomes affecting gendered physiology, or how many so-called biological parents one can potentially have (cf. the three-parent baby technique), it can be argued that past-century sexual and reproductive ethics needs a “queer injection” simply to be able to compute contemporary debates (cf. Björklund and Dahl 2020), as cis- and heteronormativity have in fact confused our understanding of the human condition. Wahlert and Fiester (2012, iii–iv) define the aims of queer bioethics as placing sexuality and gender identity at the core of ethical discussions brought about by advances and renegotiations of normality, centralizing the voices and needs of the so-called less powerful, challenging the status quo and presumptive legitimacy of the normative, and challenging LGBTQI + complacency in the face of injustice. To me, this is a perfect alignment with core feminist promoted by Donchin and Purdy. Finally, yet importantly, Wahlert and Fiester (ibid.) call for queer bioethics to be further developed into a moral theory, a project far exceeding the scope of this paper but to which project this paper aims to contribute in its own right. To give an example of the moral theory developmental needs around AMR, its framework needs to have the ethical imagination to better encompass multispecies perspective but also, it must detect the specific needs of marginalized groups in stratified health, such as lack of basic health services adding to the compound risks of extensively drug-resistant *Shigella sonnei* in the UK among men having sex with men (Charles et al. 2022), as sexual health has always been and continues to be riddled with gendered, racialised, politicised and class-based meanings (Hanley 2022).

But how could justice challenge giving priority to human ways of being, knowing and living (cf. Cañada et al. 2022; Hoppe 2020; Whitworth 2018)? That because humans are physically separate or separable from other species and non-human nature, and because humans are supposedly unique from all other species (due to capacities that justify agency), humans are therefore more important than other species or more-than-human environments (Celermajer et al. 2021, 220)? The latter notion builds on the sentience argument, crucial for defining the framework for justice; that is, making sentience a requirement for moral worth and agency. To frame the problem a bit differently, the argument that human species and its related interests should take priority over others either directly or indirectly due to agency, can be questioned with the notion of flat ontology, following Bruno Latour’s (1999; 2005) seminal work, introducing a notion of reality highly influential to feminist theory of new materialism (Barad 2007). Yet in a flat ontology stance, if all objects have the same degree of agency, what will happen to moral responsibility based on agency usually assigned to humans only? It would seem counter-intuitive, if not morally flawed in consequence, to except moral responsibility from more-than-human beings like microbes or environments, and similarly. Not to go into the full philosophical debate here, an important differentiation is to make a distinction between moral significance and moral responsibility (cf. Hayler 2022). So instead of determining ontological differences between humans and more-than-humans, we need a framework that determines ontology not based on essentialist view of being, assigning moral worth through taxonomies of sentience for example, but based on affective ability between objects, including humans. Thus, even cost-benefit thinking about justice as striking a “fair balance of benefits and burdens among those affected”, sometimes also conceptualized in terms of “harm to others” could be useful, proving that human exceptionalism bias in removed, unable to factor in let alone alleviate more-than-human harms (Cañada et al. 2022, 6). Thus, an answer to be given in more detail elsewhere, to this burning ethical questions for AMR is not to abandon justice as a principle but to recalibrate the framework by relationality following Donchin (2001), in which the central concern is a space that can be occupied by different human and nonhuman actors in a context-dependent manner. Further, this nuance is based on the idea that the same practices or the value of different forms of life cannot always be considered on the same terms (cf. Cañada et al. 2022, 8).

## A queer feminist posthumanist framework for vulnerability: antibiotic vulnerabilities

A queer feminist perspective to justice, attuned to both social and more-than-human justice, is crucial to show, how seemingly unified stakeholders, such as power structures within groups of human networks or similar more-than-human networks, have intersecting, layered vulnerabilities that align with existing social justice issues reproduced for example, by ability/embodiment, species, gender and sexuality. In such structures, reproduced through social practice, those already marginalized are at higher risk to become vulnerableized (cf. Tremain 2021), such as sex workers. For example, Salome Manuya and colleagues (2022) have shown how with the threat of AMR and entrenched gender disparities, sex workers are vulnerable to greater surveillance, stigmatization, and criminalization “to control both infections and women”. This also harks back to the lacuna of queer positioning in AMR frameworks, including mapping out specific needs and vulnerabilities for queer sex workers (cf. Charles et al. 2022; Yingwana 2022).

In AMR, there are vulnerabilities associating with injustice that follow documented patterns of oppression (e.g. Sariola et al. 2022) but also novel ways in which it deepens marginalization (Dalton 2023). Particularly gendered effects of AMR bioethics are unmapped, even though working with microbes has already shown that for example, technologies used in the management of HIV filter and subsequently reflect and reaffirm — on the level of the body — global issues related to unequal access to advanced biomedical technologies and care (McKnight 2022), and that reviving and extending work on health inequalities is important to provide evidence on the distribution of ‘need’ for antibiotics and prevent worsening inequalities through reductions in prescribing mandated by national policies (Will 2018). In mapping out these vulnerabilities and especially in seeking to eradicate them, new theoretical approaches are needed not to reiterate moral harms created by cis- and heteronormativity. I suggest queer vulnerabilities as one of its tools.

Queer vulnerabilities are my original analytical tool (Sudenkaarne 2019, 2020, 2021), combining Florencia Luna’s (2009, 2018) layered concept of vulnerability with Wahlert and Fiester’s (2014, 56) queer bioethics inventory (QBI). The current formulation consists of four layers of queer vulnerabilities focusing on kinship, intimacy, agency, and ethical sustainability. I will discuss them with the particular interest of bridging a lacuna in AMR research in relation to justice and vulnerability.

When evaluating the layer of troubled kinship, the analysis can focus on asking whether the case in question honors the diversity of families and relationships across and within the LGBTQI + population, or alternatively whether it prioritizes heterosexual marriage or the heteronormative family of origin. Does the case omit, exclude, or dismiss important characters—such as partners, lovers, or caregivers? To detect queer vulnerabilities of interrogatory intimacy, analysis can begin by asking if the case has implicitly or explicitly made value judgments about types of sexual relationships. Further, the scenario of the case may conflate “safe” or “safer” sex with monogamy or abstinence. The case can also function as a type of bioethical voyeurism, overly scrutinizing the sexual lifestyle choices of queer persons beyond their clinical or ethical relevance, as heteronormative discourses have a long history of an assumed entitlement, or even mandate, to scrutinize the intimate lives of queer persons. In these interrogations, queer people are encouraged into intimacy by the medical staff, but that intimacy is volatile, ambivalent—indeed, interrogatory (Sudenkaarne 2019, 2021).

The layer of queer agency invites analysis of whether the case patronizes the LGBTQI + individuals involved by pitying (or overly sentimentalizing) the queer subject. Vulnerabilities under this layer can be established by asking whether the queer roles of the LGBTQI + people in the case are stereotypes or overgeneralizations. Has the case infantilized the queer parties? It is also important to note whether both queer and non-queer subjects are treated as equally important and valid. Moreover, another crucial aspect of agency is the right to nondisclosure: it is important to reflect on whether the case respects the queer person’s choice and rationale to remain closeted or protective about queer health information. In terms of embodied agency and disability/crip interests, it is pivotal that nonnormative bodies are appreciated as legitimate, appropriate, and neutral. Queer agency is often misunderstood or not readable within the available epistemological paradigm, which defaults to pathology, resulting in problems of erasure and invisibility in clinical encounters, systems, and policies (Sudenkaarne 2019, 2021).

My fourth layer of queer vulnerability is the layer of ethical sustainability. Crucially, an analysis of this layer begs the question of whether there is a heteronormative value hierarchy in the case that is given priority over others. Does the case allow itself to be “dequeered” and still have ethical or clinical relevance? If not, does the queer nature of the case justify or disqualify it as worthy of legitimate study? Finally, a pivotal factor in establishing ethical sustainability is to decide whether or not unsympathetic and immaterial details about queer subjects have been included, resulting in bias against them. Although all of the four layers are to be used in evaluating ethical sustainability in the sense of just treatment, this layer offers particular insights for moral theory. However, the notion of ethical sustainability exceeds traditional research ethics approaches. They often limit themselves to solely dealing with issues of research conduct, although these are also important, as addressed by the third question under this layer. Moreover, I encourage the casting of a critical eye on how uninterruptedly ethical sense-making flows from queer subjects to non-queer subjects—what does the method of queering or dequeering reveal not only about the medical-ethical relevance of the case, but also about how ethical evaluations of care practices and outcomes are informed by cis-, hetero-, and transnormativity (Sudenkaarne 2019, 2021)?

Queer vulnerabilities are layered in the sense that they do not consider LGBTQI+ people to be similarly and categorically vulnerable; instead, queer people can be rendered vulnerable in various ways. With queer vulnerabilities, no single standard or ideal exists, and there are multiple factors of vulnerability, which are all deeply related to context and not essential properties of LGBTQI+ people. Queer vulnerabilities consist of dispositions: possibilities of being harmed, mistreated, or exploited, with varying probabilities. Crucially, intersectional analysis should can highlight queer privileges within LGBTQI+—for example how racialized transgender persons are rendered vulnerable in terms of reproductive justice differently than cis gay men even though both remain subjected to queer vulnerabilities caused by cis- and heteronormativity (cf. Honkasalo 2018; Leibetseder 2018).

Thinking with queer vulnerabilities, a topic for further research is considering queer vulnerabilities in relation to antibiotic vulnerabilities in the context of AMR. Antibiotic vulnerabilities are a concept by Eleanor E. MacPherson (2022) and colleagues, building on the premise that while policy makers grapple to reduce the overuse of antimicrobial medicines to stem the rise of antimicrobial resistance, insufficient attention has been paid to how this applies to low-resource contexts. Based on their fieldwork in context of extreme scarcity in rural Malawi, they found that care became centered around provision of an antimicrobial. Revealing the multiple ways in which provision of antimicrobials can impact care, they (2022, 2632) note that as targets for optimal antimicrobial prescribing take a more central role in global policy, close attention is required of the ramifications for the delivery of care to ensure that efforts to stem resistance do not undermine the goal of improved health “for all”. In a queer feminist posthuman framework, “all” should include more-than-human stakeholders (McPherson et al. 2022, 2632).

MacPherson and her team (2022) suggest to me a multilayered connection between vulnerability and AMR. One the hand, ineffectiveness of antimicrobials will have significant ramifications for health systems, where treatment of vulnerable patients is likely to become more challenging, as well as the treatment of everyday illness where next-line antibiotics are unavailable (McPherson et al. 2022; Jasovský et al. 2016). They remind us that, stemming resistance to antimicrobials – particularly antibiotics – has become a global priority as the decreasing efficacy of these previously taken-for-granted substances renders visible the vulnerability of our health, economics and security systems globally.

Yet the proposed framework to address AMR – the WHO’s Global Action Plan (2015) – has primarily framed AMR as a problem of excess, centring overall reduction in antibiotic use as the main goal (McPherson et al. 2022, 2632; Sariola et al. 2022).

Ushered in by ‘pharmaceuticalisation’ since the 1990’s, the shift from prevention of disease to pharmaceutical intervention and treatment (e.g. Bell & Figert 2015), McPherson and colleagues (2022, 2632) observe that over time AMR discourse has come to consider not only people themselves as vulnerable to resistant infections but antibiotics too as vulnerable resources. As such, the expansion of stewardship programmes that aim to protect medicines foregrounds certain forms of vulnerability. To them (ibid.), an exploration of the intersection of the assumed and enacted vulnerabilities of both people and medicines has the potential to steer the course of stewardship, especially if these vulnerabilities can be traced as dynamic, emergent of systemic and programmatic features and amplified in contexts of scarcity. Further, they argue that these dynamics co-constitute a form of care that both responds to and creates antibiotic vulnerabilities. Dimensions of care in chronic scarcity present a scenario in which optimising antibiotic use will require not only stable availability of essential medicines but also a re-ordering of the system around provision of quality care rather than of medicines. So practices of case management can be understood to both respond to and create antibiotic vulnerabilities. (McPherson et al. 2022, 2632.)

Based on their analysis, McPherson and colleagues (2022, 2643) conclude that recognizing vulnerability often provokes a need to protect. Antibiotics are often portrayed as vulnerable, for example in awareness campaigns which call on publics and professionals to protect these medicines (e.g. Langdridge et al. 2019). Patients are also vulnerable in the face of drug resistance, but moreover, they (ibid.) show that by tracing out the ways in which antibiotics are deployed in case management in resource constrained settings, patients are also vulnerable to receiving sub-standard care that is provided through an unhelpful antibiotic. Similarly to previously discussed queer vulnerabilities critique against cis- and heteronormative bias in medical ethics, McPherson and colleagues (2022, 2643) show that both patients and health workers police the boundaries of expected scripts of ‘care’, delivered without explanation, bolstering a scenario that perpetuates patient vulnerabilities. As one the outcomes, they (ibid., see also Chandler 2019; Will 2018) claim that stewardship interventions aiming to reduce and restrict antibiotic prescribing are a misfit for low-income contexts and could have significant unintended consequences for patients and health workers.

McPherson and colleagues (2022) showcase the importance of context in AMR stewardship. Moreover, context-specific analysis must also inform conceptualization of AMR as a bioethical phenomenon as imaginable policies and management practices are directly related, albeit not often analyzed as such in drafting policies, to the framework in which this conceptualization takes place. While antibiotics should warrant a level of protectionism, it is important to stay open to recognizing layered vulnerabilities while simultaneously staying open to more-than-human justice orientation: thinking along the lines of protectionism – neither in relation to microbial realms such as pathogens ─ nor in terms of human health exceptionalism, but also not of the environment alone ─ does not suffice for ethically sustainable approaches to AMR.

McPherson and colleagues (2022, 2641) conclude that while ensuring a stable drug supply including antibiotics is vital, it must be accompanied by wider changes that enable quality care to be performed and received beyond antibiotic-oriented case management. They (ibid.) propose context-specific programs be designed not only to protect vulnerable antibiotics, and patients vulnerable to antibiotic resistance, but also to protect patients from systems that have come to be organized around antibiotics, resulting in case management that leaves patients vulnerable to insufficient care (ibid.). Building on their proposition that considering multiple dimensions of antibiotic vulnerabilities has conceptual promise as it can at once bring to the frame the vulnerability of antibiotics, people and systems, for future research is to consider cases of AMR, gender and sexuality, such as queer sex workers situated in a specific geographical location while also informed by global conditions of stratified health, for context-specific programs and specific antibiotic vulnerabilities.

## Conclusion

On a conceptual level on antibiotic vulnerabilities and in a queer feminist framework, it is important to consider the following parallel. While vulnerability is both the quality of antibiotics and people in AMR, protection against the latter can in fact be weaponized (see Fiester 2015) against vulnerabilities of the former as antibiotic-focused care is insufficient. Similarly, cis- and heteronormativity in bioethics can threaten basic human rights of LGBTQI + people and allow exclusion from bioethical principles (Sudenkaarne 2021). In a queer feminist framework for vulnerability in AMR, it becomes detectable how cis- and heteronormativity in bioethical practices, systems, and policies can infringe the flow of ethical sustainability. At its worst, this infringement becomes intense human suffering, causing the loss of queer lives, and establishing the need for LGBTQI + bioethics pragmatically, clinically, and morally (Sudenkaarne 2019; 2021). Further, this analysis needs to be accompanied with a more-than-human justice orientation (see Sudenkaarne and Butcher in this special issue) for firstly to acknowledge, how more-than-human actors already play an important role in AMR, and secondly, how these roles should be acknowledged in bioethical theory. Moreover, however, crucial for this analysis is not lose sight of the existing power disparities within human species. 

## Footnotes

1 These obligations, and also the language of risks, can be seen to resonate with principles, but Luna herself does not make that connection.
